# Intestinal microbiota development and gestational age in preterm neonates

**DOI:** 10.1038/s41598-018-20827-x

**Published:** 2018-02-06

**Authors:** Katri Korpela, Elin W. Blakstad, Sissel J. Moltu, Kenneth Strømmen, Britt Nakstad, Arild E. Rønnestad, Kristin Brække, Per O. Iversen, Christian A. Drevon, Willem de Vos

**Affiliations:** 1 Immunobiology Research Programme, Department of Bacteriology and Immunology, University of Helsinki, Helsinki, Finland; 2 European Molecular Biology Laboratory, Heidelberg, Germany; 3 Department of Pediatric and Adolescent Medicine, Akershus University Hospital and Institute for Clinical Medicine, Campus Ahus, University of Oslo, Nordbyhagen, Nordbyhagen, Norway; 4 Department of Nutrition, Institute of Basic Medical Sciences, Faculty of Medicine, University of Oslo, Oslo, Norway; 5 Department of Neonatal Intensive Care, Division of Paediatric and Adolescent Medicine, Oslo University Hospital, Ullevål, Oslo, Norway; 6 Department of Neonatal Intensive Care, Division of Paediatric and Adolescent Medicine, Oslo University Hospital, Rikshospitalet, Oslo, Norway; 7 Institute of Clinical Medicine, Faculty of Medicine, University of Oslo, Oslo, Norway; 8 Department of Haematology, Oslo University Hospital, Oslo, Norway; 9 Laboratory of Microbiology, Wageningen University, Wageningen, The Netherlands

## Abstract

The intestinal microbiota is an important contributor to the health of preterm infants, and may be destabilized by a number of environmental factors and treatment modalities. How to promote the development of a healthy microbiota in preterm infants is largely unknown. We collected fecal samples from 45 breastfed preterm very low birth weight (birth weight < 1500 g) infants from birth until 60 days postnatal age to characterize the intestinal microbiota development during the first weeks of life in preterm infants. Fecal microbiota composition was determined by 16S rRNA amplicon sequencing. The main driver of microbiota development was gestational age; antibiotic use had strong but temporary effects and birth mode had little influence. Microbiota development proceeded in four phases indicated by the dominance of *Staphylococcus, Enterococcus, Enterobacter*, and finally *Bifidobacterium*. The *Enterococcus* phase was only observed among the extremely premature infants and appeared to delay the microbiota succession. The results indicate that hospitalized preterm infants receiving breast milk may develop a normal microbiota resembling that of term infants.

## Introduction

Prematurity is a risk factor for infant morbidity and mortality^1^, and is associated with a high risk of bacterial and inflammatory diseases, such as sepsis and necrotizing enterocolitis (NEC). Preterm infants have an immature intestine with underdeveloped peristalsis, barrier function and immunity, which makes the intestine a potential source of infections and inflammation^2,3^. Several studies have shown that the intestinal microbiota in preterm infants differ from that of healthy term infants^4–11^. Common anomalies are the bloom of opportunistic and potentially pathogenic bacteria such as *Enterobacter, Enterococcus*, and *Staphylococcus*^4–7^. Many common practices, caesarean section^12–14^, antibiotic use^15^, and formula feeding^14–16^, may disrupt normal microbiota development. Moreover, studies with small groups of infants have indicated that both NEC and late-onset sepsis are associated with alterations in the microbiota^8–11^, and that the microbiota composition correlates with signs of pain and distress^17^. Modification of the microbiota by probiotics or breast milk may reduce the risk of NEC in preterm infants^18,19^. Thus, promoting a healthy microbiota development in preterm infants is important, and a key question is whether natural colonization and succession of the microbiota is possible in these infants. For rational decision-making and consideration of therapies in the future, information on the microbiota development in preterm infants is essential. Although studies on the preterm microbiota have been conducted^4–11^, they involve only a handful of infants and none show detailed microbiota succession.

To establish a defined and predictive picture of the preterm infant microbiota development, we analyzed 262 fecal samples from 45 preterm infants using deep 16S rRNA amplicon sequencing.

## Methods

### Study design and participants

The samples were collected during a randomized controlled trial conducted in 2010 at Akershus and Oslo University Hospitals, Norway. The study was approved by the Regional Committee for Medical and Health Research Ethics, Norway (Reference number: 2009/1946) and performed in accordance with the principles of the Helsinki Declaration. Written informed consent was obtained from the parents. The study was registered in Clinical Trials (registration number NCT01103219) on 12. April 2010. The trial was designed to investigate the effect of enhanced nutrient supply to very low birth weight infants (VLBW; birth weight < 1500 g) as previously described^20^.

All VLBW infants born between August 17th and December 21st 2010 in the study hospitals were eligible for inclusion. Exclusion criteria were congenital malformations, chromosomal abnormalities, critical illness with short life expectancy and clinical syndromes known to affect growth and development. Infants were included after written informed parental consent was obtained and randomized into two nutrition groups within 24 h after birth. Fifty-seven infants were eligible for inclusion but 7 infants were not included due to parental consent refusal (1), critically sick mother/sibling (3), congenital anomaly (1) and omissions during enrollment (2). Thus, 50 infants were included in the trial. Three infants died during the first weeks of life and two infants were excluded due to critical illness and congenital heart disease. Hence, 45 VLBW infants were included in the present study investigating the microbiota development. Twenty-four of these infants received enhanced nutrient supply, i.e. increased supply of energy, protein, fat, the long-chain polyunsaturated fatty acids (PUFA) and vitamin A^20^. Twenty-one infants were classified as extremely premature (EP) and 24 as moderately or very premature (MVP). Caesarean delivery was common, especially in the MVP group (Table 1).

**Table 1:** Characteristics of the extremely premature (EP) and moderately/very premature (MVP) infants.

	EP n = 21	MVP n = 24	P
GA, wk, median (IQR)	26 (25.1–27.2)	30.0 (29.0–31.2)	<0.001
BW, median (IQR)	830 (727–990)	1205 (1009–1302)	<0.001
BW z-scores, median (IQR)	−0.5 (−1.2–0.2)	−1.1 (−1.9–0.8)	<0.01
SGA, n (%)	11 (46%)	4 (19%)	0.07
Boys, n (%)	12 (57%)	17 (71%)	0.34
C-section, n (%)	12 (57%)	21 (88%)	0.04
Prenatal steroid exposure, n (%)	20 (95%)	23 (96%)	1
Apgar 1 min, median (IQR)	5.0 (3.0–7.5)	7.5 (6.0–8.8)	<0.01
Apgar 5 min, median (IQR)	7.0 (6.0–8.0)	8.5 (6.0–9.0)	0.03
Respirator days, median (IQR)	11.0 (2.5–31.5)	0 (0–2.8)	<0.001
Oxygen dependency at 36 w PMA, n (%)	10 (50%)*	0 (0%)	<0.001
Late-onset sepsis, n (%)	16 (76%)	6 (25%)	0.001
IVH grd ≥ 3, n (%)	3 (14%)	1 (4%)	0.33
NEC, n (%)	2 (10%)	0 (0%)	0.21
PDA treatment (medical/surgical), n (%)	9 (45%)	0 (0%)	<0.001
ROP (grd III/+disease), n (%)	4 (20%)*	0 (0%)	0.04

Data are presented in number (percentages) or median (IQR), *n = 20.

BW = birth weight; C-section = Cesarean section; EP = Extremely premature; GA = gestational age; IQR = interquartile range; IVH = intraventricular haemorrhage; MVP = moderate or very premature; NEC = necrotizing enterocolitits; PDA = persistent ductus arteriosus; PMA = post menstrual age; ROP = retinopathy of prematurity; SGA = small for gestational age.

Four types of antibiotics were given to the infants: aminoglycosides (gentamycin, N = 10) usually combined with ampicillin/ekvacillin (N = 26), and vancomycin (N = 6) usually combined with cephalosporin (N = 12). Ampicillin and cephalosporin were not given alone. The antibiotics were all given intravenously. Vancomycin was not given to any of the infants during the first 10 days of life.

### Fecal sample collection

Fecal samples were collected from diapers at different intervals from birth until discharge from the hospitals (up to 60 days postnatal age). The median (range) number of samples per infant was 6 (2–11). Fresh samples were collected from diapers and frozen at −80 °C until analyzed. In total 262 fecal samples were analyzed.

### 16S rRNA amplicon sequencing and pre-processing

The microbiota composition of the fecal samples was analyzed using 16S rRNA amplicon sequencing with Illumina MiSeq. DNA was extracted using the repeated bead beating method, and for sequencing we used the V1-V3 primers forward AGAGTTTGATCMTGGCTCAG and reverse GTATTACCGCGGCTGCTG. The reads were processed and analyzed using the R-package mare^21^, which utilizes USEARCH^22^ for quality filtering, clustering of reads into species-like operational taxonomic units (OTUs), and taxonomic annotation. The analysis procedure was validated using artificial microbial communities of known composition^21^. Specifically, we assessed how the observed microbiota composition depended on read length. We found that the forward reads trimmed to 100–150 nucleotides resulted in better correspondence to the actual composition in the artificial communities than using long, merged paired-end reads (Supplementary Fig. 1). Thus, we used only the forward reads, which we truncated to 150 nt, to remove low-quality bases at the end of the reads. The 150nt reads were quality-filtered. All sequences representing less than 0.001% of the total reads were eliminated, as rare reads are likely to contain errors or chimaeras. The reads were OTU-clustered for richness analysis. The OTUs were not annotated, as OTU-clustering is a potential source of taxonomic errors. Instead, we taxonomically annotated the reads. After quality filtering, we had on average 68 000 reads per sample, ranging from 23 to 282 000. The samples with <100 reads were meconium samples, in which very low abundance of bacteria is expected, and therefore we considered the low number to be biologically relevant. For the meconium samples we obtained on average 26 000 (23–98 570) reads. We included also 14 empty samples as negative controls in the sequencing run. The number of reads obtained for these samples varied between 0 and 822 (median = 37, mean = 164). We compared the composition observed in the meconium samples with <1000 reads to that in the negative control samples (Supplementary Fig. 2). Four of the meconium samples had control-like compositions high in lactobacilli, suggestive of contamination. These few meconium samples were unlikely to alter the results, and they were kept in the analysis.

### Statistical analyses

The statistical analyses were conducted using the R package mare^21^, which implements tools from the R package vegan^23^. Principal coordinates analysis (PCoA), which summarizes the multivariate microbiota data into a few variables, was conducted using Bray-Curtis dissimilarities with the R-package vegan^23^. Associations between the most abundant bacterial groups with the host factors postmenstrual age, postnatal age, and birth mode (vaginal/cesarean) as well as antibiotic courses (aminoglycoside or vancomycin) were tested using generalized additive mixed models (GAMM), with negative binomial error distribution (function gam in the package mgcv^24^), subject ID as the random variable, and the number of reads as the offset.

### Data availability

The data are available in Supplementary Table 1. The DNA sequences will be submitted to European Nucleotide Archive (http://www.ebi.ac.uk/ena).

### Term infant samples

To compare the microbiota development in the premature infants to the microbiota in term-born healthy infants, we included previously reported 16S rRNA–based microbiota composition data from a Dutch infant cohort^25^ in which 9 fecal samples were collected from 187 infants during the first 15 postnatal weeks (total 665 samples).

## Results

### Development of microbiota in preterm infants

**Figure 1:**
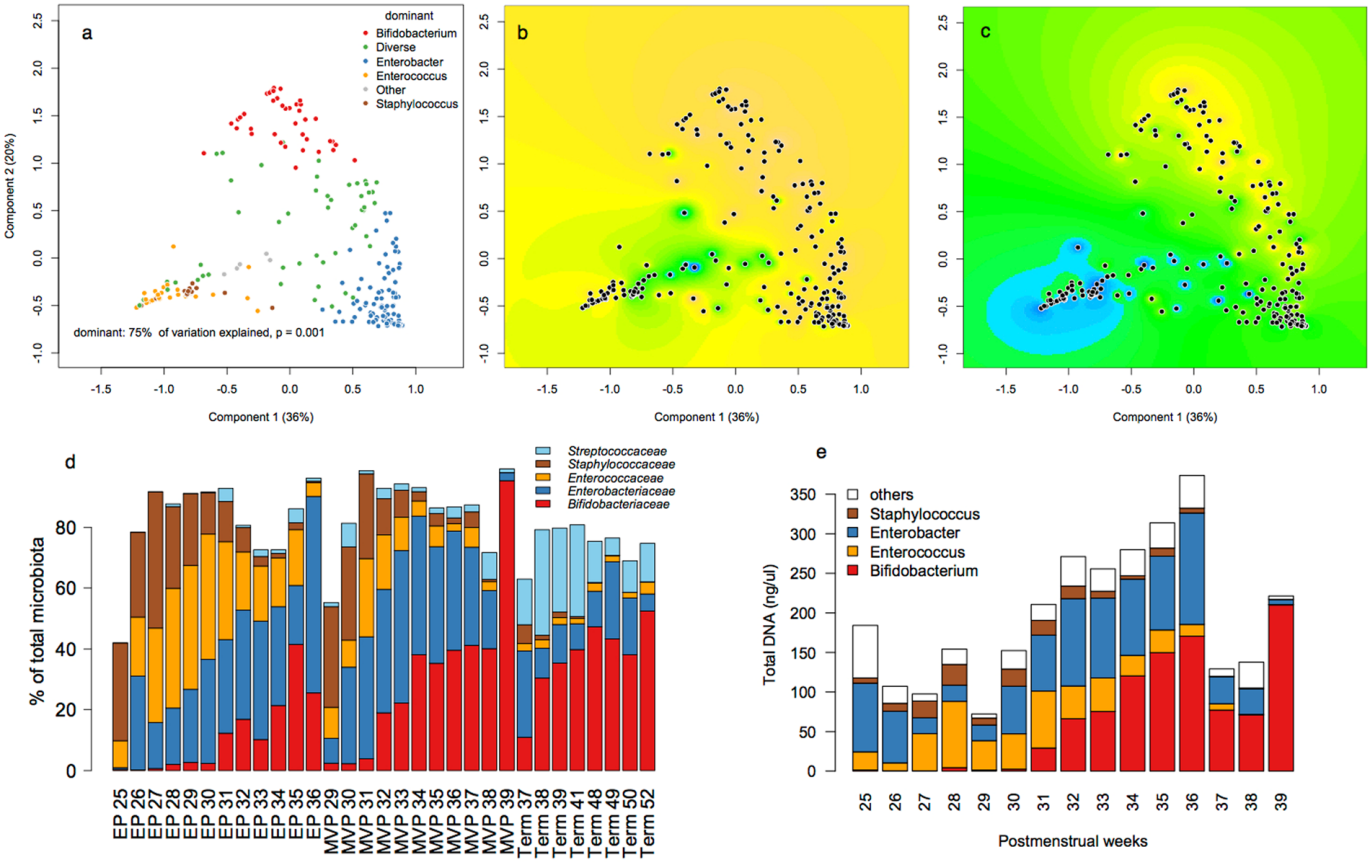
Microbiota composition in the fecal samples of preterm infants. Principal coordinates analyses (PCoA, Bray-Curtis dissimilarities) (**a**–**c**). The axes represent PCoA component scores, showing the two most important gradients differentiating the microbial communities. Each circle represents a microbial community, colored in panel (**a**) by the dominant organism in the community (>50% of all reads). Background color corresponds to: (**b**) postnatal age of the infant at the time of fecal sample collection; (**c**) postmenstrual age of the infant at sample collection. Blue background indicates low values, green intermediate, and orange high values. Average relative abundances of the dominant families in extremely premature (EP), moderately or very premature (MVP), compared to term infants from a Dutch cohort, at different postmenstrual ages (weeks)^25^ (**d**). Average total DNA concentration by postmenstrual age, divided to bacterial taxa based on their relative abundances in the 16S rRNA data (**e**).

The intestinal microbiota composition in the 45 premature neonates was longitudinally followed and was characterized by strong dominance of only a few organisms. Typically one of four genera, *Bifidobacterium, Enterobacter, Staphylococcus* or *Enterococcus*, represented >50% of the reads in a given sample (Supplementary Fig. 3, Fig. 1a). The infants often switched from one pattern of microbiota (dominating organism) to another within days (Supplementary Fig. 3). The overall microbiota composition was strongly associated with postnatal age (Fig. 1b, R^2^ = 0.07, p = 0.001 in permutational multivariate ANOVA) and even more clearly with postmenstrual age (Fig. 1c, R^2^ = 0.13, p = 0.001). After adjusting for postmenstrual age, postnatal age explained only 2% of the variation (p = 0.002). With increasing postmenstrual age, the infants progressed from *Staphylococcus-Enterococcus*-dominated composition to *Enterobacter* and finally towards *Bifidobacterium*-dominated microbiota (Fig. 1c).

We compared the observed microbiota composition at different postnatal ages in the EP and MVP infants to healthy term-born infants. The microbiota composition in the preterm infants progressed towards the composition observed in the term-born infants, depending on gestational age (Fig. 1d); the microbiota development in the EP group appeared to lag behind that of the MVP group (Fig. 1d), but the development was matched in both groups when considering the postmenstrual age. In fact, when comparing the MVP and the term infants at the same postmenstrual age, it appeared that the microbiota of the MVP infants progressed faster towards a strongly *Bifidobacterium*-dominated composition (Fig. 1d).

To assess the development in bacterial load with age, we measured the total DNA concentration in the fecal samples (Fig. 1e). For further insight, the DNA yield was subdivided to different bacterial taxa based on their relative abundances. The amount of DNA in the samples was fairly stable until 30 weeks postmenstrual age, after which it began to increase, possibly due to an increase in the absolute abundance of bifidobacteria. Simultaneously, the absolute abundance of staphylococci and enterococci appeared to decline. After 36 weeks postmenstrual age, there appeared to be a decline in DNA yield, as bifidobacteria became dominant and all other bacteria declined.

**Figure 2:**
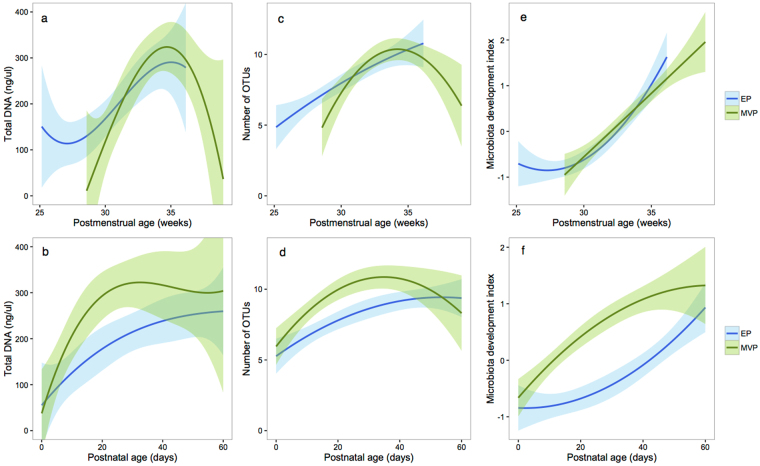
Development of the microbiota with postmenstrual (**a**,**c**,**e**) and postnatal (**b**,**d**,**e**) age. Association between postmenstrual and postnatal age of the infant and total DNA concentration (**a**,**b**), microbial richness (**c**,**d**), and development index (**e**,**f**, sum of the two first principal components in Fig. 1) among infants born before (extremely premature, EP), and at/after gestational week 28 (moderately or very premature, MVP). The trend lines show the best-fit (second polynomial) and the shaded areas show 95% confidence intervals.

Overall, the DNA concentration increased significantly with postmenstrual age (Fig. 2a, p = 0.03, explaining 14% of the variation), and with postnatal age (Fig. 2b, p = 0.0002, 7% of the variation), indicating an increase in total bacterial abundance, and therefore active replication. The DNA concentration was not dependent on birth mode (p = 0.8, 0.1% of variation).

Simultaneous with the increase in DNA concentration, richness of the microbiota increased with postmenstrual age (Fig. 2a, p = 0.0014, explaining 20% of the variation), and with postnatal age (Fig. 2b, p = 0.02, 7% of the variation). Richness was not associated with birth mode (p = 0.19, 1% of the variation). The microbiota development with age was captured by the sum of the score on the first two principal coordinates (Fig. 1a–c). We used the PCoA score sum as the development index. This index was positively associated with postmenstrual age (Fig. 2c, p < 0.0001, 35% of the variation), but after adjusting for postmenstrual age, the association with postnatal age was not significant (Fig. 2d, p = 0.4, 1% of the variation). The development index tended to be lower in vaginally born infants (p = 0.07, 1% of the variation).

### Microbiota development in phases

**Figure 3:**
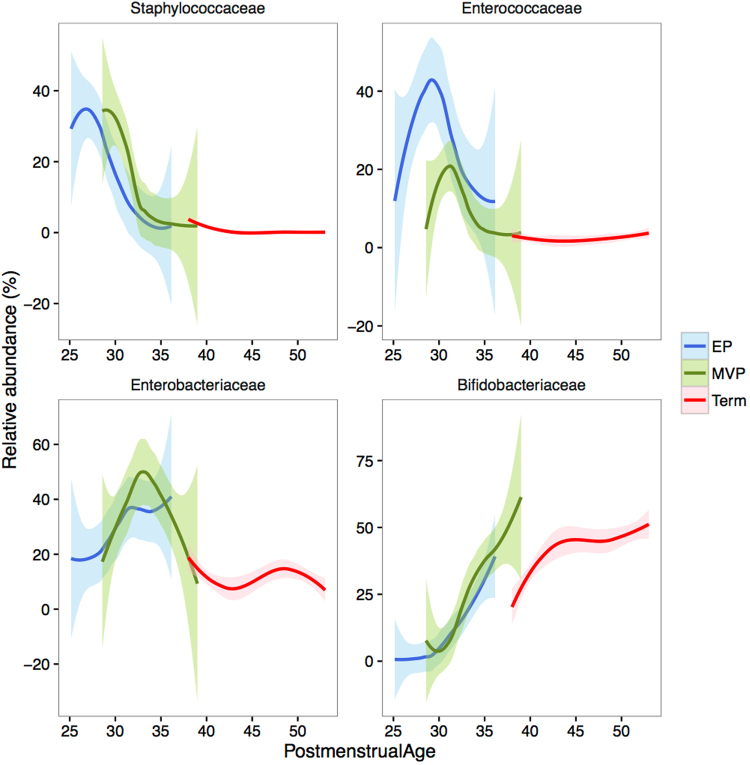
Development of the most abundant bacterial genera in premature and term-born infants with respect to postmenstrual age. The trend lines show the best-fit (second polynomial) and the shaded areas show 95% confidence intervals.

A detailed analysis of the most abundant genera revealed that the development of the microbiota composition was not random, but followed a pattern depending on postmenstrual age (Fig. 3). The gestational age at birth appeared to have little influence on the microbiota development, as the EP, MVP, and healthy term-born infants followed largely the same course of development. In the first 50 weeks postmenstrual age, there were four successional phases.

Phase 1 was *Staphylococcus* dominance, which peaked between 25 and 30 weeks postmenstrual age and ended by 35 weeks postmenstrual age, and therefore did not occur in term-born infants (Fig. 3a). The abundance of *Staphylococcus* was significantly associated with postmenstrual age (p < 0.0001), which explained 20% of the variation in *Staphylococcus* abundance, while postnatal age explained an additional 8% of the variation (p < 0.0001). Vaginally born infants had a higher abundance of *Staphylococcus*, although birth mode explained only 1% *Staphylococcus* abundance (p = 0.004). Individuality covered 8% of the variation in *Staphylococcus* (p = 0.016).

Phase 2 was *Enterococcus* dominance, peaking at 30 weeks and ending by 35 weeks postmenstrual age (Fig. 2b). Postmenstrual age explained 3% of the variation in the abundance of enterococci (p = 0.0001), and postnatal age explained 2% (p = 0.002). Birth mode was not significantly associated with the relative abundance of *Enterococcus* (p = 0.74). *Enterococcus* abundance showed high individuality, subject ID explaining 30% of the variation (p < 0.0001).

Phase 3 was *Enterobacteriaceae* dominance (mainly genus *Enterobacter* in the preterm infants), peaking on average at 35 weeks postmenstrual age (Fig. 2c). The term-born infants showed only the declining part of this successional phase. There appeared to be a modest secondary bloom of enterobacteria at 45–50 weeks postmenstrual age. Among the preterm infants, the timing of the *Enterobacter* peak showed extensive individual variation and consequently the postmenstrual age explained only 1% of the variation (p = 0.47), and postnatal age explained 4% (p = 0.22). Vaginal birth was associated with lower abundance of *Enterobacter* (p = 0.01, 1% of variation explained).

Phase 4 was characterized by a high abundance of *Bifidobacterium*, which began to develop gradually after 30 weeks postmenstrual age. Postmenstrual age explained 11% of the variation in *Bifidobacterium* abundance (p < 0.0001), and postnatal age explained 5% (p = 0.39). There was considerable individuality in *Bifidobacterium* abundances, ID explaining 24% (p < 0.0001), while birth mode explained 1% (p = 0.22).

#### Associations between microbiota and antibiotic treatments

**Figure 4:**
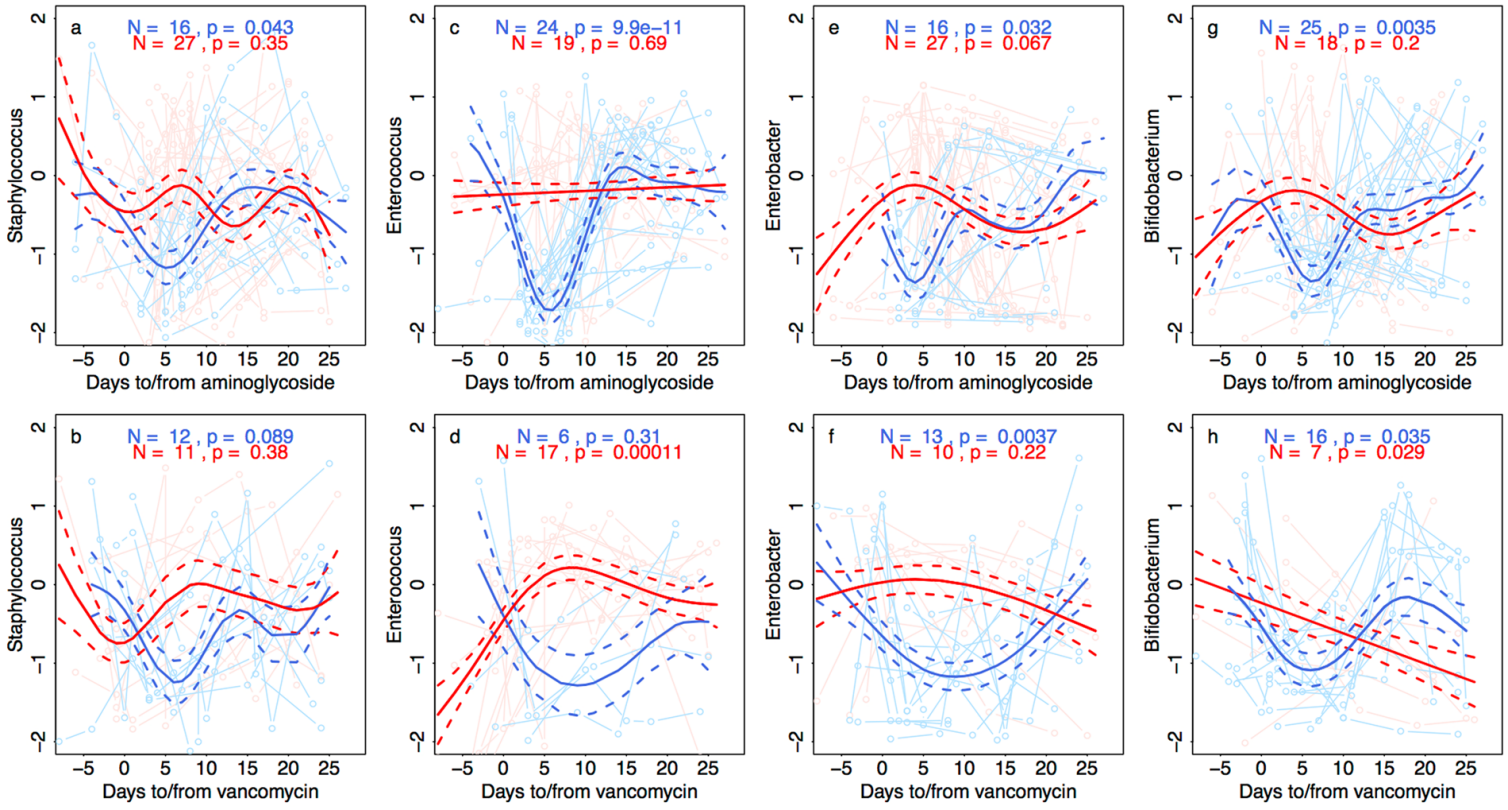
Effect of antibiotic treatments on the most abundant bacterial genera. Deviation from expected relative abundance of each genus, based on age of the infant and birth mode, is shown in relation to timing of the antibiotic courses. Consistently negative values after the beginning of the antibiotic course (between days 1 and 10) are interpreted as a response to the antibiotic, and the infants are categorized as responders (blue) or non-responders (red). Number of infants in each category and the p-values of the GAMM models are shown.

To assess the effect of antibiotic treatments on the microbiota, we focused on the period before and after antibiotic courses, and estimated the effect of aminoglycoside and vancomycin antibiotics after adjusting for postmenstrual age, postnatal age, and birth mode. The responses to the antibiotics were generally temporary and the microbiota appeared to recover within days (Fig. 4, Supplementary Fig. 4). Furthermore, only a subset of the infants responded to the antibiotics, showing lower than expected values during the first 10 days after the beginning of the antibiotic, while others either showed no response or even the opposite, increasing response.

In terms of total DNA, microbiota richness, and microbiota development index, 37% of the infants responded temporarily to aminoglycoside (Supplementary Fig. 4a,c,e). The rest either did not respond or showed a temporary increase in total DNA and development index (Supplementary Fig. 4a,e). Vancomycin treatment was associated with a decline in total DNA and development index in 52% of the infants, but richness was not significantly affected (Supplementary Fig. 4b,d,f).

Individual bacterial taxa did not always respond according to the total DNA response (Fig. 4). *Staphylococcus* and *Enterobacter* followed the same pattern as total DNA, showing a short-term response to aminoglycoside in 37% of the infants and to vancomycin in ca. 50%. *Enterococcus* showed a dramatic short-term decline in response to aminoglycoside in 56% of the infants, and increased in response to vancomycin in 74% of the infants. Bifidobacteria declined in 58% of the infants temporarily in response to aminoglycoside treatment. Vancomycin treatment was associated with either a short-term reduction of bifidobacteria, or a slower but persistent reduction.

#### Microbiota composition and sepsis incidence

**Figure 5:**
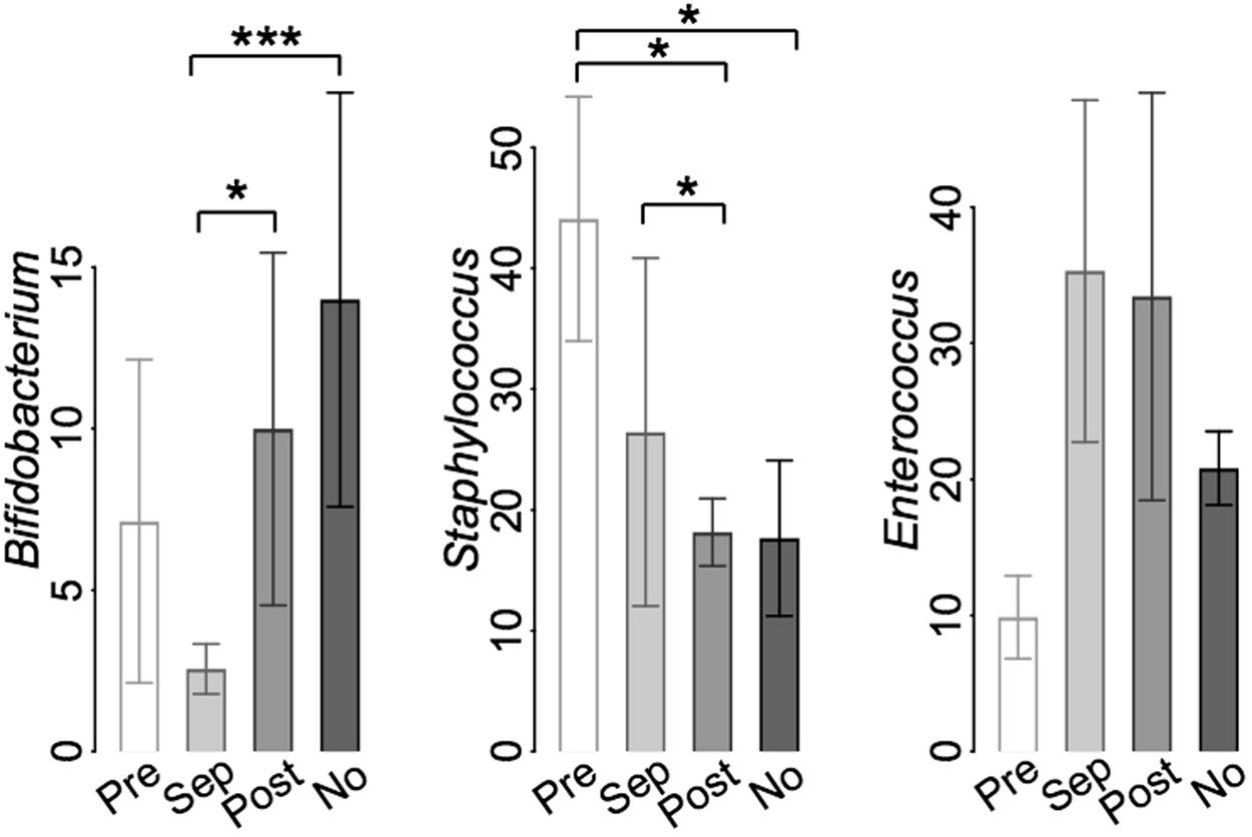
Relative abundances of *Bifidobacterium*, *Staphylococcus*, and *Enterococcus* before (“Pre”), during (“Sep”), and after (“Post”) sepsis, compared to infants with no sepsis (“No”).

Fourteen cases of sepsis in 10 infants were documented during the study period with fecal samples collected within five days of diagnosis. Based on blood cultures, the causative organisms of sepsis in the analyzed cases were always staphylococci. *Staphylococcus* was more abundant before sepsis than after (Fig. 5), and in 7/14 cases, *Staphylococcus* was dominant in the fecal samples either before or during sepsis. During sepsis, the dominant organism in the fecal samples was *Enterobacter* in five cases, *Enterococcus* in five cases, and *Staphylococcus* in four cases (Supplementary Fig. 2). Sepsis did not occur in infants with *Bifidobacterium* as the dominant organism; the abundance of *Bifidobacterium* was significantly reduced during sepsis (Fig. 5).

## Discussion

We followed the development of the intestinal microbiota longitudinally in a cohort of 45 prematurely born infants from birth to discharge from hospital up to 60 days after birth. Frequent sampling enabled the detailed construction of individual developmental patterns, and characterisation of the microbiota succession. Our results show that the promotion of normal microbiota development in prematurely born infants is possible in a hospital setting, because many infants progressed to a *Bifidobacterium*-dominated composition which is characteristic of healthy term-born non-hospitalized infants. A major obstacle for the microbiota development among the extremely premature infants was the overgrowth of *Enterococcus* spp, which seemed to inhibit the normal succession by several weeks in some cases.

We found maturity, indicated by postmenstrual age, to be a major determinant of the microbiota development, and particularly of the ability of bifidobacteria to reach dominance. This has previously been observed in term and premature infants^26,27^. Regardless of the gestational age at birth, infants began to proceed towards a *Bifidobacterium*-dominated composition, an indicator of healthy microbiota in infants, after postmenstrual week 30. The results show that although the extremely premature infants had significantly more co-morbidities and required more medical interventions than the moderately premature group, both premature groups followed the same microbiota development pattern. This suggests that physical maturity is a very strong determinant of microbiota development in neonates.

In our cohort, infants with a disrupted microbial development, showing a dominance of aerobic cocci and reduced abundance of bifidobacteria, were at increased risk of sepsis. A dominance of *Enterobacter* spp. or other Proteobacteria, and the absence of bifidobacteria have previously been associated with risk of necrotizing enterocolitis and sepsis in preterm infants^8–11^; in our cohort *Enterobacter* was abundant in some sepsis cases, but also in many cases not developing sepsis.

As the microbiota development is dependent on infant maturity, particularly the extremely premature infants are at risk of delayed microbiota development and may require therapeutic interventions. Supplementation with bifidobacteria or bifidogenic prebiotics has been shown to increase the abundance of bifidobacteria in preterm infants, to reduce the abundance of clostridia and enterobacteria, and to decrease signs of distress^17,28,29^. Antibiotics may have a harmful effect on the microbiota development^30^. In line with this, we observed temporary changes in microbiota composition following antibiotic courses. The microbiota appeared to recover within 2–3 weeks. Importantly, the response to antibiotics depend on the susceptibility of the bacteria to the drug. For example, vancomycin-resistant enterococci prevail in hospital environments, and may prevent a beneficial response.

Unlike most previous studies, our cohort included many infants with a fairly normal microbiota development, achieving high abundance of bifidobacteria. All infants in our study received human milk from the first days of life. Breast milk feeding is known to increase the abundance of bifidobacteria in infants^31,32^. The increase in bifidobacteria was achieved also in caesarean-delivered infants, demonstrating that normal microbiota development is possible even in these prematurely born, hospitalized, caesarean-delivered infants. This suggests that the recently suggested practice of vaginal seeding^33^, i.e. swabbing the neonate with the mother’s vaginal fluids, which has been criticized as potentially risky^34^, is unnecessary if breast milk (from mother or donor) is given.

Our results suggest that normal-like microbiota development in preterm infants in the hospital setting is possible, particularly among moderately premature infants receiving breast milk. In the extremely premature infants, development appeared to be disrupted by persistently increased abundance of *Enterococcus*. Future research on interventions to improve the microbial colonization in extremely premature infants and infants with patterns indicating abnormal developmental patterns, could improve short- and long-term outcomes.

## Electronic supplementary material

Supplementary Information

Supplementary Table 1
