# Supplementary material for: Intestinal microbiota development and gestational age in preterm neonates
Source: Sci Rep. 2018 Feb 6;8:2453. doi: 10.1038/s41598-018-20827-x (PMC5802739; doi:10.1038/s41598-018-20827-x)
Supplement: Supplementary file 1 — Supplementary Information [file 41598_2018_20827_MOESM1_ESM.pdf]

Supplementary Information

Intestinal microbiota development and gestational age in preterm neonates

Katri Korpela, Elin W. Blakstad MD, Sissel J. Moltu, Kenneth Strømmen, Britt Nakstad, Arild E. Rønnestad, Kristin Brække, Per O Iversen, Christian A. Drevon, Willem de Vos

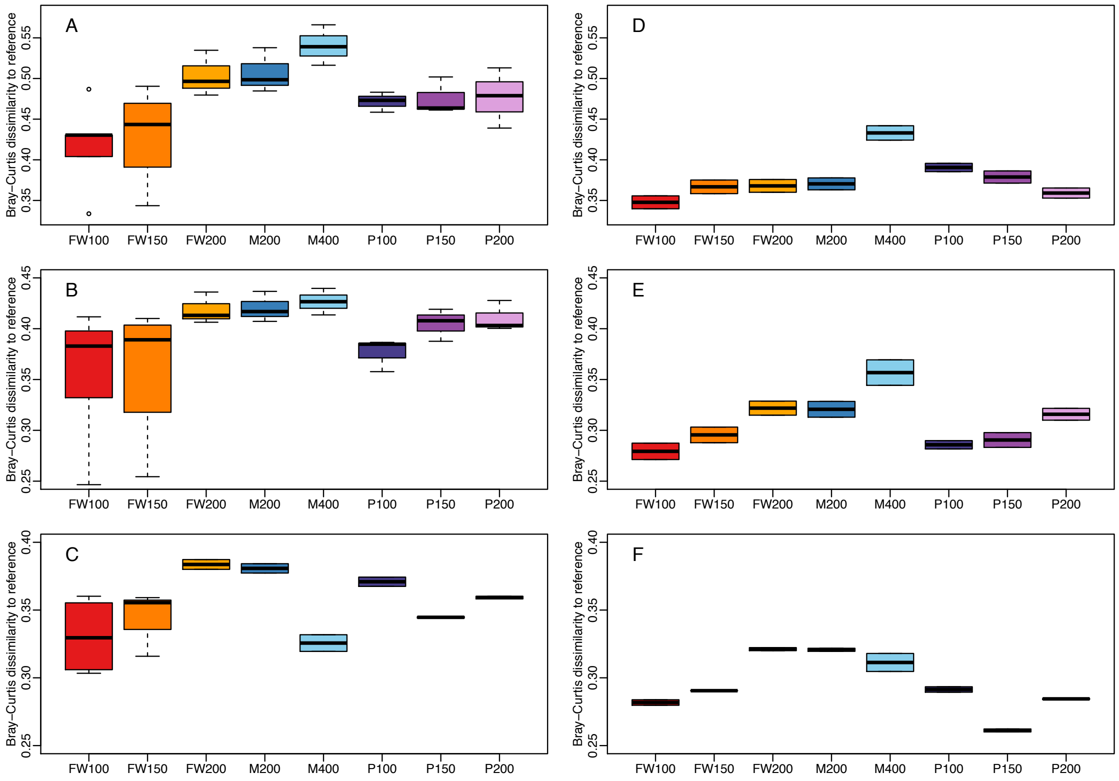

Supplementary Figure 1. Comparison of 8 different sequence processing protocols: forward reads only, trimmed to 100 nucleotides (FW100), 150nt (FW150), or 200nt (F200), merged paired-end reads trimmed to 200nt (M200), or 400nt (M400), pooled but not merged forward and reverse reads, each forward-reverse read pair treated as two separate reads, trimmed to 100nt (P100), 150nt (P150), or 200nt (P200). Three artificial microbial communities were subjected to two different PCR protocols (1-step PCR, panels A-C, and 2-step PCR, panels D-F) and sequenced in two different MiSeq runs and two different HiSeq runs. Panels A and D show results for community 1 (17 species), panels B and E for community 2 (54 species), and panels C and E for community 3 (9 species). The observed microbiota compositions are compared to the expected composition using Bray-Curtis distance; the lower the distance, the more similar the observed and expected compositions are.

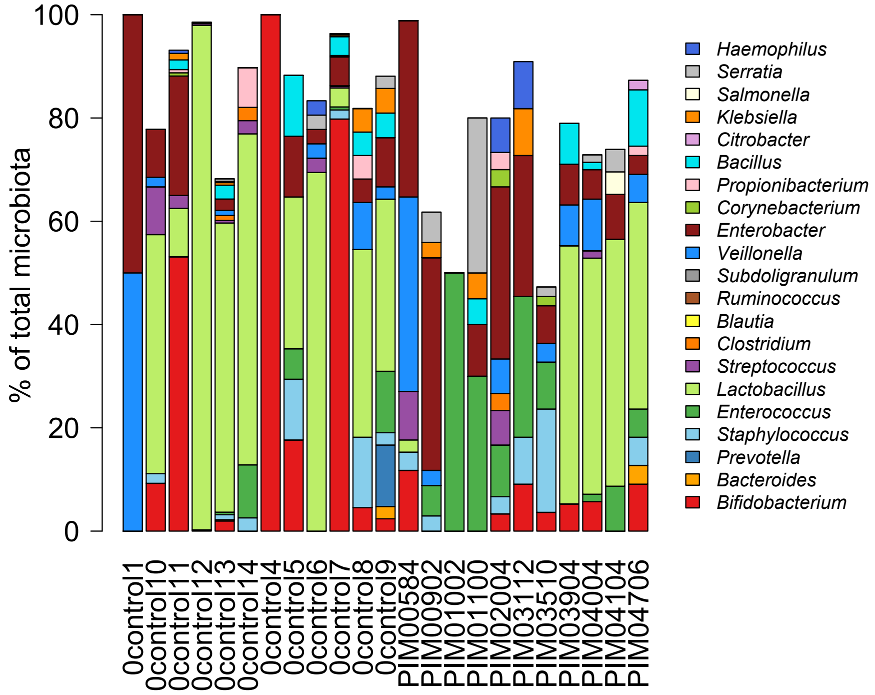

Supplementary Figure 2. Observed microbiota composition in negative control samples (control) and in meconium samples with <1000 reads (PIM).

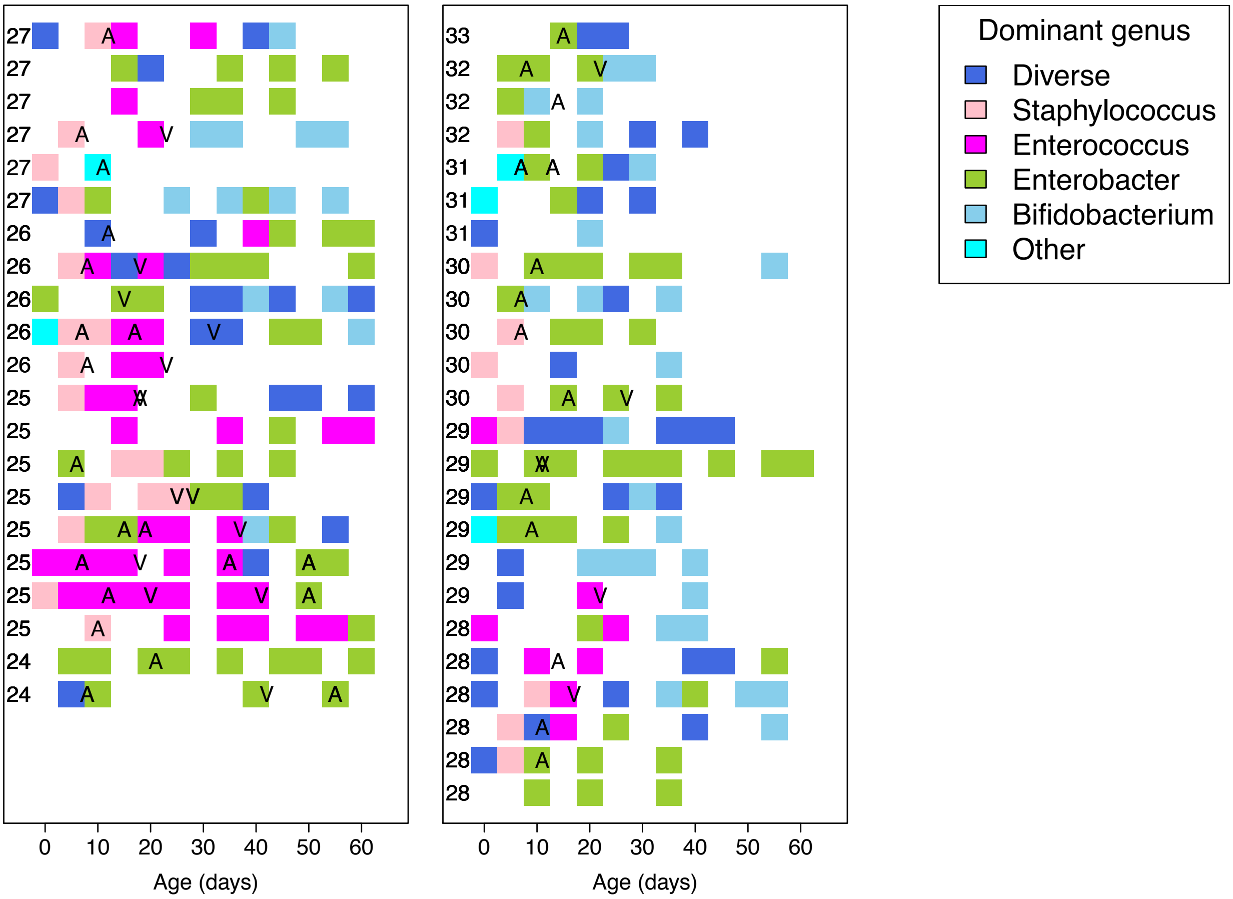

Supplementary Figure 3. Microbiota samples from 45 infants at indicated time points. Each row represents one infant. Panel A shows infants born before gestational week 28 (extremely premature infants, EP); panel B shows infants born at or after week 28 (moderately or very premature infants, MVP). Gestational age at birth is given in the first column, and Caesarean-born infants are indicated in red. The columns represent sample collection times as postnatal days (±4 days). The letters indicate antibiotic treatment. A = aminoglycoside, V = vancomycin. The colors indicate the dominant genus in the sample, representing > 50% of total abundance. The sample was classified as “diverse” when no single genus was dominant.

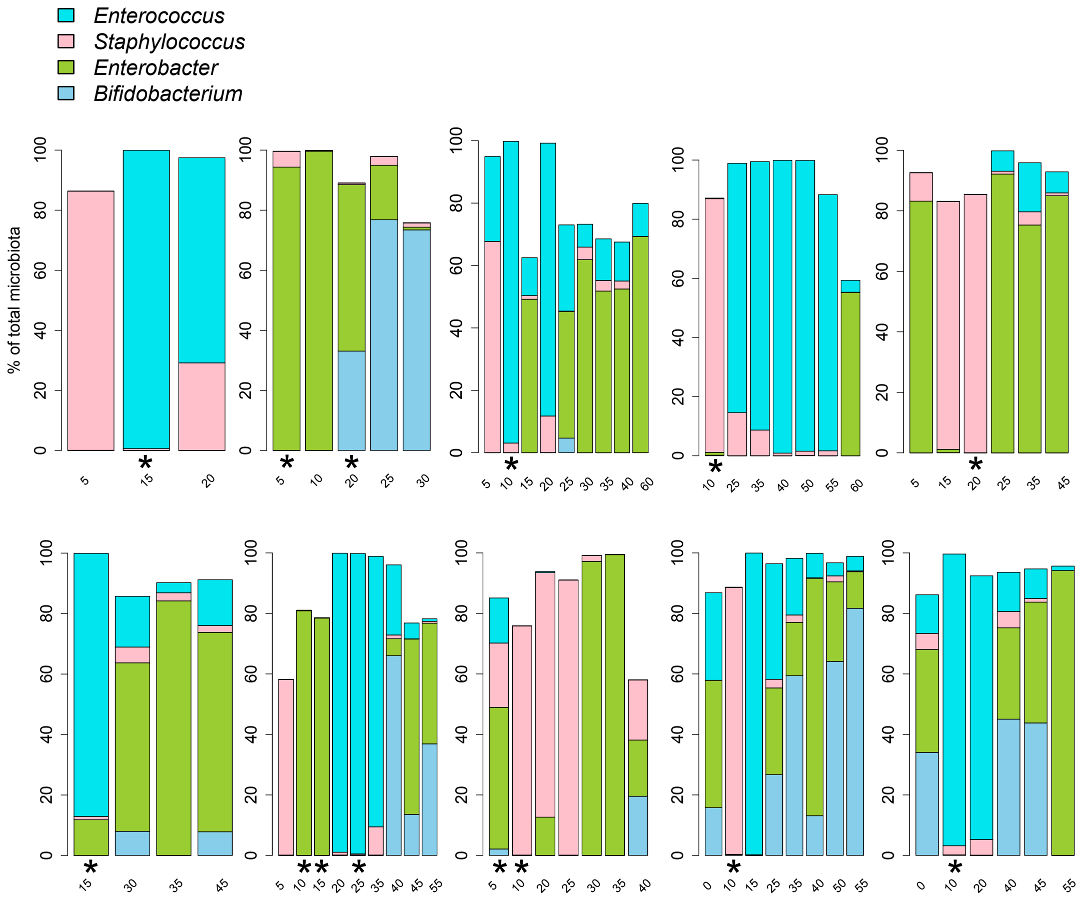

Supplementary Figure 4. Relative abundance of the dominant organisms in infants diagnosed with sepsis during the study period. Age of the infant (postnatal days) is indicated on the x-axis. Timing of sepsis is indicated by asterisks. Blood cultures showed that staphylococcus caused all sepsis cases.

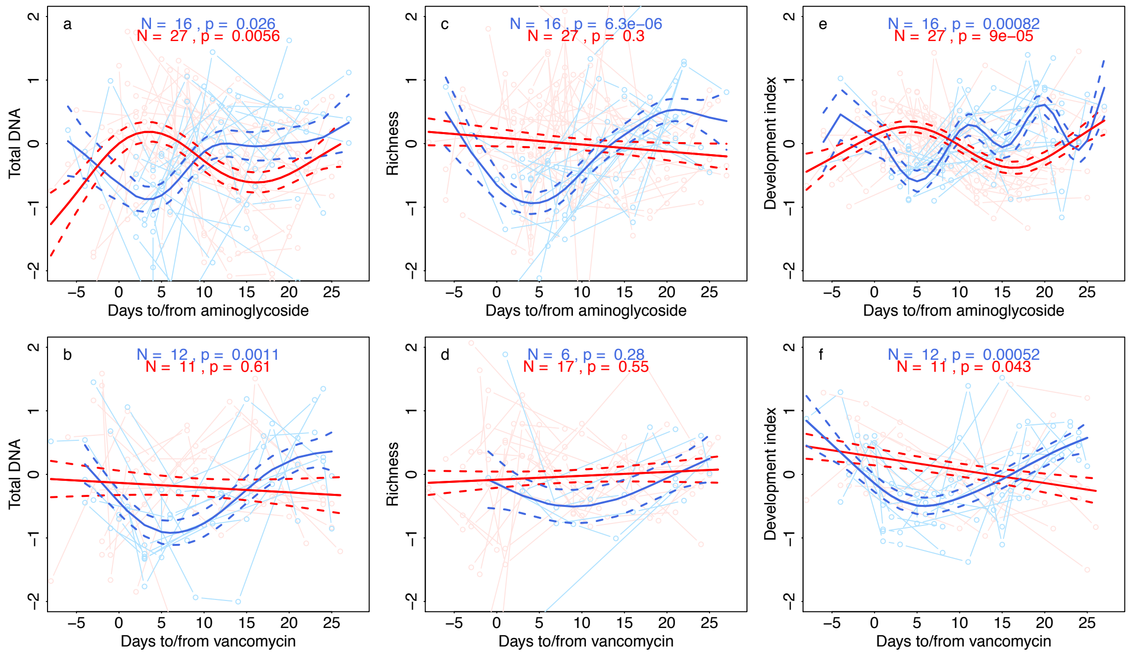

Supplementary Figure 5. Effect of antibiotic treatments on total DNA concentration, microbial richness and development index. Deviation from expected values, based on age of the infant and birth mode, is shown in relation to timing of the antibiotic courses. Consistently negative values after the start of the antibiotic course (between days 1 and 10) are interpreted as a response to the antibiotic, and the infants are categorized as responders (blue) or non-responders (red). Number of infants in each category and the p-values of the GAM models are shown.
